# Upregulated LINC00922 Promotes Epithelial-Mesenchymal Transition and Indicates a Dismal Prognosis in Gastric Cancer

**DOI:** 10.1155/2022/1608936

**Published:** 2022-04-11

**Authors:** Xiaojing Chen, Lanxin Hu, Xinrui Mao, Haoran Chen, Yuchen She, Hao Chi, Hao Zeng, Lu Guo, Yunwei Han

**Affiliations:** ^1^Department of Oncology, The Affiliated Hospital of Southwest Medical University, Luzhou, China; ^2^Department of Clinical Medicine, Southwest Medical University, Luzhou, China; ^3^Department of Ophthalmology, The Affiliated Hospital of Southwest Medical University, Luzhou, China

## Abstract

**Background:**

LINC00922 has been found to promote epithelial-mesenchymal transition (EMT) in a variety of tumors. But its functions in gastric cancer (GC) remain unclear. We attempt to investigate the correlation between LINC00922 and GC via bioinformatics analysis, in vitro and in vivo experiments.

**Methods:**

TCGA and GTEx databases were utilized to obtain the RNAseq and clinical data of GC, and then, identified the correlation of LINC00922 with patients' clinicopathological characteristics and prognosis. GSEA and GO/KEGG enrichment analyses were performed to explore the potential functions or signaling pathways that LINC00922 participated in GC. Infiltration levels of immune cells were employed by ssGSEA algorithm, and then Wilcoxon rank sum test was applied to analyze their correlations with LINC00922. Scratch and transwell assays were conducted to detect the invasion and migration abilities of GC cells. Western blot was performed to explore the expression level of EMT-related proteins. Furthermore, we constructed the xenograft tumor model and metastatic tumor model in nude mice to explore the effect of LINC00922 downregulating on metastasis of GC cells in vivo.

**Results:**

Compared with normal tissues, LINC00922 was highly expressed in GC tissues and positively correlated with poor prognosis. The correlation existed between LINC00922 and immune infiltration in GC. Downregulation of LINC00922 inhibited the EMT process of GC cells. In addition, both in vitro and in vivo experiments showed that LINC00922 affects the invasion and migration abilities of GC.

**Conclusions:**

LINC00922 promotes the migration, invasion, and EMT in GC and has the potential to be used as a prognostic biomarker and therapeutic target for GC.

## 1. Introduction

Gastric cancer (GC) is the fifth most common malignancy and the fourth leading cause of cancer-related death, and more than one million cases newly diagnosed as GC worldwide in 2020 [1]. Early-stage GC can be treated surgically and patients have a high survival rate, while the outcome of patients with advanced-stage is not satisfactory [2]. The age-standardized 5-year overall survival (OS) rates for GC are low in many countries, ranging from 20% to 40% [3]. Recent breakthroughs in omic technologies have permitted unprecedented levels of cancer sequencing and characterization, revealing novel growth pathways and disease genetic drivers [4]. Although various treatment methods have advanced, the prognosis of GC patients remains poor [5]. Therefore, searching for biomarkers for early diagnosis and exploring the molecular carcinogenesis of GC are urgently required to improve the prognosis of this cancerous disease.

Long noncoding RNAs (lncRNAs) are RNA transcripts that are longer than 200 nucleotides in length and have no or limited protein-coding potential [6]. lncRNAs can be classified into long intergenic ncRNA (lincRNA), antisense lncRNA, sense lncRNA, and intronic lncRNA, of which lincRNA and antisense lncRNA are the most common types [7]. lncRNAs are involved in regulating a variety of biological functions and some of these functions depend on the intracellular localization: the regulation of lncRNAs in the nucleus involves chromatin interactions, transcription, and RNA processing, while lncRNAs in the cytoplasm are involved in regulating mRNA stability, translation, and cell signaling [8]. Compared to transcripts encoding proteins, lncRNAs usually exhibit a higher degree of temporal and spatial specificity [9]. Therefore, identification of differential lncRNA expression profiles in GC would aid in the diagnosis and could serve as an effective therapeutic target [10].

Studies have illustrated a diverse range of biological processes of lncRNAs in the regulation of the occurrence and progression of various tumors, including GC [11]. Among them, evidence has confirmed the involvement of lncRNAs in the regulation of gene expression associated with GC metastasis. For example, lncRNA SNHG11, as an oncogene, mediates the proliferation and migration of GC cells by binding miR-184, then weakening the inhibitory effect of miR-184 on CDC25A [12]. Tumor-infiltrating immune cells influence drug response in GC patients [13, 14]. So, the relationship between lncRNAs and tumor immunity has received much attention. For example, lncRNA SNHG15 increases PD-L1 expression by suppressing miR-141, which in turn promotes resistance to immune response and immune escape of GC cells [15]. The involvement of lncRNAs in immune regulation is currently considered to be complex, and many key immunoregulatory lncRNAs have not yet been identified [16].

LINC00922 (long intergenic nonprotein coding RNA 922), also known as lnc-LALC, is a lincRNA with a chromosomal localization of 16q21, and a full length of 1776 bp containing nine exons. In normal human tissues, LINC00922 was detected in lung, spleen, whole blood, and testis with high expression levels [17]. Previous studies reported that the prognostic model consisting of six lncRNAs (SNHG12, MAFG-DT, ASMTL-AS1, LINC02321, LINC01322, and LINC00922) has shown the prognostic value of OS for bladder cancer patients, which reflects the prognostic potential of LINC00922 [18]. During the epithelial-mesenchymal transition (EMT) process, epithelial cells lose cell polarity and cell-cell adhesion and then acquire a mesenchymal stem cell phenotype with migratory and invasive properties [19]. EMT is considered as the key event in the process of tumor metastasis [20]. Previous studies showed that LINC00922 can regulate the proliferation, migration, invasion, and EMT process of hepatocellular carcinoma cells as a sponge of miR-424-5p [21]. Similarly, LINC00922 promotes EMT by binding miR-361-3p in ovarian cancer [22]. In addition, LINC00922 can also activate the Wnt signaling pathway by promoting methylation of promoter NKD2, which in turn promotes invasion, migration, and EMT of breast cancer [23]. The above results suggested LINC00922 can be involved in regulating the EMT process as miRNA sponges or methylation regulators.

Currently, although LINC00922 has been found to play an oncogenic role in various tumors, its functions in GC are still lacking. In this study, we investigated LINC00922 expression and its correlations with clinicopathological features and clinical outcomes of GC patients. Then, its impact on the EMT process and metastasis of GC was tested. Furthermore, we discussed the potential association of LINC00922 with tumor immune infiltrating cells. We aimed to provide new insights into the prognostic biomarker and treatment strategies for GC patients.

## 2. Materials and Methods

### 2.1. Data Processing and Differential Expression Analysis

We extracted RNAseq data of TCGA and GTEx database in transcripts per million reads (TPM) format from the UCSC Xena database (https://xenabrowser.net/datapages/), which had been uniformly processed by the Toil process and then log2-transformed for further analysis [24]. Furthermore, we obtained RNAseq data and clinical data in TCGA-STAD project level 3 HTSeq-FPKM format from TCGA database (https://portal.gdc.cancer.gov/), then FPKM format RNAseq data were converted to TPM format and proceeding log2-transformed. Finally, we collected a total of 407 GC projects, including 375 GC tissues and 32 adjacent normal gastric mucosa tissues. Among them, 27 pairs of cancer tissues and paired adjacent normal tissues were contained. The baseline data of GC patients were shown in Supplementary Table S1. Then, we analyzed the relationship between LINC00922 expression and the clinicopathological characteristics of GC through the Chi-square test, Wilcoxon rank sum test, and logistic regression analysis methods.

### 2.2. Survival Analysis

We performed receiver operating characteristic (ROC) curve analysis of LINC00922 expression in GC samples. We used the “survival analysis” module to analyze the correlation of LINC00922 with OS and disease-free survival (DFS) in GC through the GEPIA2 tools [25]. With the cutoff-high (50%) and cutoff-low (50%) values as the expression threshold, the high and the low expression group were divided. And log-rank test was used for hypothesis testing. We used Kaplan-Meier Plotter database (http://kmplot.com/analysis/) to evaluate the relationship between LINC00922 and postprogression survival (PPS) in GC patients [26]. Furthermore, we used this database to perform prognostic analyses based on LINC00922 expression levels in GC cohort of specific immune cell subsets. We used univariate and multivariate Cox regression to analyze the prognostic factors related to GC, including the clinicopathological features and the expression level of LINC00922.

### 2.3. KEGG and GO Analysis

We obtained the top 100 genes coexpressed with LINC00922 in GC from the GEIPA2 database and used the R package ClusterProfiler [27] to conduct KEGG pathway analysis and GO enrichment analysis.

### 2.4. Gene Set Enrichment Analysis (GSEA)

We obtained RNAseq data in Level 3 HTSEQ-Counts format from TCGA-STAD project and removed the control/normal items. Based on the median expression level of LINC00922, patients were divided into high and low expression groups. GSEA enrichment analysis and visualization were played via the cluster Profiler R package [27]. For enrichment analysis results, while the absolute value of normalized enrichment score (NES) > 1, *P*.adj < 0.05, and the false discovery rate (FDR) < 0.25, the enriched signaling pathways were considered to be statistically significant.

### 2.5. Expression of LINC00922 and Analysis of Immune Infiltration in GC

The immune infiltration analysis of GC was performed through the single sample gene set enrichment analysis (ssGSEA) in the GSVA R package, and the degrees of the infiltration of 24 immune cells were quantified based on gene expression profiles [28, 29]. Wilcoxon rank sum test was used to analyze the correlation between the degree of immune cell infiltration and LINC00922 expression level.

### 2.6. Materials

Fetal bovine serum was purchased from Serana (Germany), and RPMI-1640 medium was purchased from Hyclone (Beijing, China). Puromycin and polybrene were purchased from Beyotime (Shanghai, China). Total RNA extraction kits were purchased from Tiangen (Beijing, China). Reverse transcription kits and quantitative real-time PCR kits were purchased from Vazyme (Nanjing, China). LINC00922 siRNA lentivirus (si-LINC00922) and negative control (NC) lentivirus (viral titer: 8 × 10^8^TU/ml) were purchased from GenePharma (Shanghai, China). The sequences of siRNA were shown in Supplementary Table S2. The 24 well plates and the matrigel (356231) for transwell assay were purchased from Corning Incorporated (USA). The antibodies include GAPDH (60004-1-Ig), E-Cadherin (20874-1-AP), N-Cadherin (22018-1-AP), and vimentin (10366-1-AP) antibodies were purchased from Proteintech (Hubei, China).

### 2.7. Cell Culture and Lentiviral Transfection

In this experiment, human GC cell lines MGC-803, MKN-45, and immortalized human gastric epithelial cell lines GES-1 were selected for culture. All cells were kept in a humidified incubator at 37°C with 5% CO_2_. Cells were cultured in RPMI 1640 medium supplemented with 10% fetal bovine serum and 1% penicillin-streptomycin. When cells reached about 80% confluence, they were subcultured. And for lentiviral transfection, an appropriate amount of polybrene and siRNA lentivirus was added to transfect target cells. After 48 hours of transfection, puromycin was added for screening. After 7 days of continuous screening, stably transfected cell lines were obtained.

### 2.8. Quantitative Real-Time PCR (qPCR)

Total RNA in the cells was extracted by using trizol reagent. And after RNA was reverse-transcribed into cDNA, qPCR was accomplished using SYBR Green real-time PCR kit, where a 20 *μ*l reaction system was set up according to the protocol. The gene expression level was calculated by the 2^-∆∆CT^ method. The GADPH served as the internal reference gene. The primers used in this experiment were synthesized by Sangon Biotech (Shanghai, China), and the primer sequences were shown in Supplementary Table S2.

### 2.9. Transwell Migration/Invasion Assay

The wells were first coated with 100 *μ*l matrigel. When cells reached 80-90% confluence, they were digested. After centrifugation, resuspension, and density adjustment (1.0 × 10^4^ cell/*μ*l), 200 *μ*l cell suspension was seeded to the upper chamber, and 600 *μ*l 1640 medium containing 10% fetal bovine serum was added to the lower chamber. After 48 hours of incubation, the medium was removed and then cells were fixed in absolute alcohol. After fixing, chambers were taken out of the plate, and absolute alcohol was removed. Chambers were then dyed in crystal violet. The inner side of the membrane was wiped with cotton swabs, and the chambers were turned upside down to get dry. Finally, cells were observed with an inverted microscope. Five randomly selected fields of view per sample were counted, and data obtained were then analyzed. The procedure for migration assay was the same as the invasion assay except that matrigel was not used.

### 2.10. Western Blot

When the confluence of the cells reached 80% and grew well, total proteins were extracted. The protein concentration was determined via the BCA method. After electrophoresis, proteins were transferred to polyvinylidene fluoride (PVDF) membranes. PVDF membranes were then blocked with 5% skimmed milk for 2 hours and incubated overnight at 4°C with primary antibodies. On the second day, membranes were incubated with secondary antibodies for 2 hours. All bands were detected using a chemiluminescence ECL kit and quantified by ImageJ software. The GADPH served as the internal reference protein.

### 2.11. Construct Xenograft Tumor Model and Metastatic Tumor Model in Nude Mice

Male athymic BALB/C nude mice (5 weeks old, weight 16-20 g) were purchased from TengXin Biotechnology Co. Ltd. (Chongqing, China). Then, they were placed in a temperature-controlled environment (20-22°C) with a relative humidity of 50-60% and circulated in light and dark for 12 hours. Nude mice could freely use standard laboratory food and drink tap water. And they were cared for in accordance with the National Institutes of Health (NIH) guidelines for Laboratory Animals Care. All animal experiments were approved by the Institutional Animal Care and Treatment Committee of Southwest Medical University (Luzhou, China). 24 nude mice were randomly divided into four groups (6/group). Xenografts were modeled by subcutaneous injection of MKN-45 cells (1.0 × 10^6^ cell/ml) into the right side of the nude mice. Tumor volumes were measured at two-day intervals beginning on day 7 and calculated as: 0.5 × length × width^2^. The nude mice were sacrificed after 21 days, the tumor tissues were fixed, and the expression levels of E-cadherin, vimentin, and N-cadherin proteins were analyzed by IHC. To establish metastatic tumor models, 100 *μ*l MKN-45 cells (5.0 × 10^6^ cell/mL) were injected into nude mice (3/group) through the caudal vein. The nude mice were sacrificed after 30 days, and the lung and liver tissues were fixed and analyzed for metastasis by H&E staining.

### 2.12. Immunohistochemistry and H&E Staining

Tumors and organs were fixed over 24 hours inside a 10% neutral buffered formalin solution, then embedded in paraffin and cut into 2-4 *μ*m thick slices. Hematoxylin and eosin (H&E) was used to stain the lung and liver tissue sections, which were then blindly examined by two pathologists. Antihuman E-cadherin, vimentin, and N-cadherin antibodies were used to stain tumor tissue slices, and the slices were viewed under the microscope (Olympus BX43, Tokyo, Japan) at ×400 magnification. E-cadherin, vimentin, and N-cadherin proportions were tallied in five randomly selected fields for each sample, then, mean values were determined.

### 2.13. Statistical Analysis

The raw data collected in the experiment were statistically processed using SPSS 23.0 software and GraphPad 9.0. The comparison between two groups was done using an independent sample *t*-test, and the comparison between numerous groups was done with a one-way analysis of variance. In all statistical analyses, *P* < 0.05 was the significance threshold (ns, *P* ≥ 0.05; ^∗^, *P* < 0.05; ^∗∗^, *P* < 0.01; ^∗∗∗^, *P* < 0.001).

## 3. Results

### 3.1. The Expression of LINC00922 in Human Cancers

**Figure 1:**
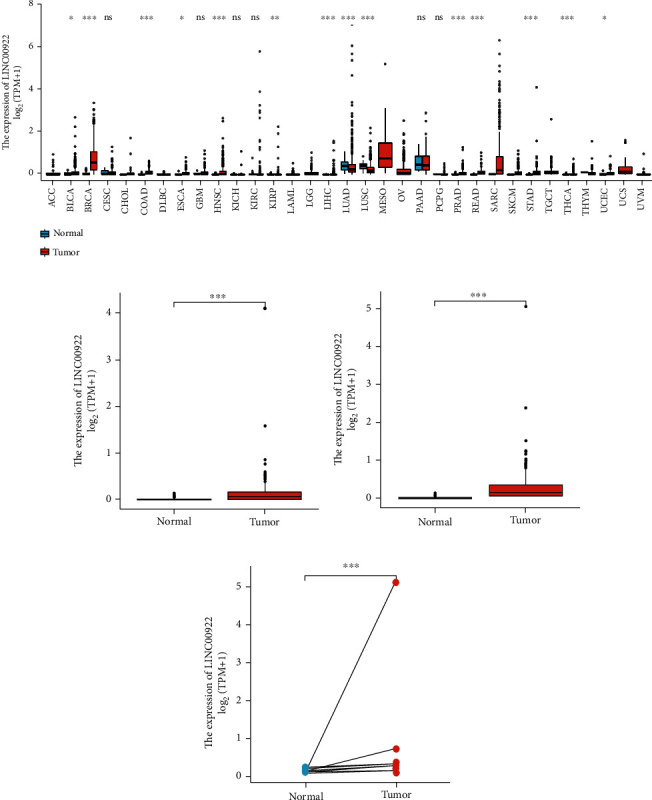
Wilcoxon rank sum test was used to detect the LINC00922 expression levels in human cancers. (a) LINC00922 expression levels in different cancer types from the UCSC Xena database. (b) The differential expression of LINC00922 in adjacent normal tissues and GC tissues of GTEx combined with the TCGA database. (c) The differential expression of LINC00922 in GC tissues and adjacent normal tissues of the TCGA database. (d) The differential expression of LINC00922 in GC tissues and adjacent normal tissues.

We analyzed the expression of LINC00922 in human cancers by analyzing the RNAseq data from UCSC Xena database. The results showed that significantly higher expression of LINC00922 in most tumor tissues compared with matched normal tissues, and the GC tissues were no exception (Figure 1(a)). In order to further clarify the expression differences of LINC00922 in GC, we first compared the expression of LINC00922 in 210 samples of adjacent normal tissues and 414 samples of GC tissues obtained from GTEx and TCGA databases. We found that LINC00922 was significantly overexpressed in GC tissues (Figure 1(b)). Then, we analyzed the expression level of LINC00922 in 375 samples of GC tissues and 32 samples of adjacent normal tissues in the TCGA database and found that LINC00922 was highly expressed in tumor tissues (Figure 1(c)). We also analyzed the expression of LINC00922 in 27 pairs of tumor tissues and matched adjacent normal tissues, which showed higher expression of LINC00922 in tumor tissues (Figure 1(d)).

### 3.2. The Correlations between LINC00922 and Clinicopathological Characteristics of GC

The clinicopathological characteristics of GC patients were shown in Supplementary Table S1. We collected 375 primary GC samples with clinical and RNAseq data from the TCGA database, then divided them into high (*n* = 188) and low expression groups (*n* = 187) in accordance with the median value of relative expression level of LINC00922. Then, we analyzed the relationship between LINC00922 and clinicopathological characteristics of GC by the Chi-square test, except that Wilcoxon rank sum test was used for age. The results showed that the expression of LINC00922 was correlated with T stage (*P* = 0.004), race (*P* = 0.022), histological type (*P* = 0.001), and histological grade (*P* = 0.049) in GC patients (Supplementary Table S1).

**Figure 2:**
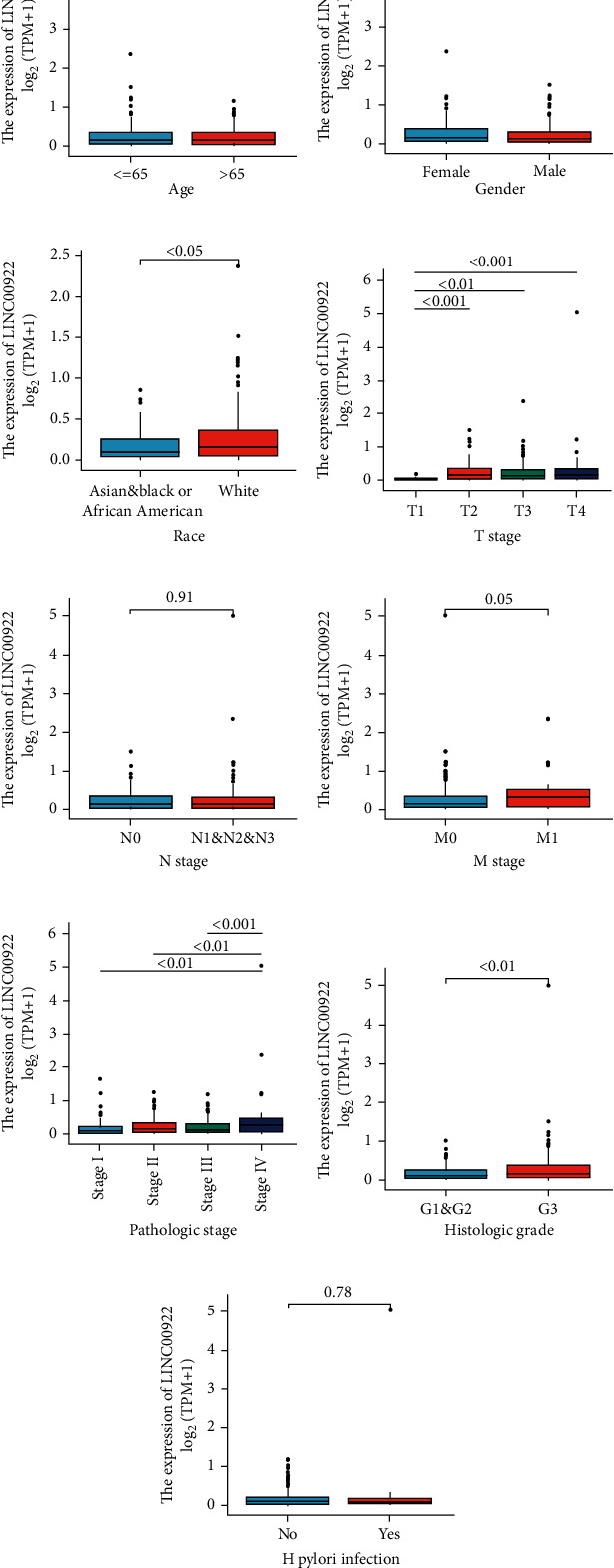
Wilcoxon rank sum test was used to compare the relationship between the expression level of LINC00922 and age (a) of GC patients in TCGA database. And did the same analysis with gender (b), race (c), T stage (d), N stage (e), M stage (f), pathological stage (g), histologic grade (h), and H pylori infection (i).

Next, Wilcoxon rank sum test was used for further analysis. The results indicated that the expression level of LINC00922 was correlated with race, T stage, M stage, histological grade, and pathological stage of GC patients, and the difference was statistically significant (*P* < 0.05). In addition, the correlation of LINC00922 with age, gender, N stage, and H pylori infection was not statistically significant (Figure 2). In addition, through the logistic regression analysis, LINC00922 expression in GC was found to be positively associated with T stage, RACE, histologic grade, and histological type, suggesting that patients with high LINC00922 expression are more likely to progress to a later stage than those with low LINC00922 expression (Table 1).

### 3.3. The Prognostic Significance of LINC00922 in GC

**Figure 3:**
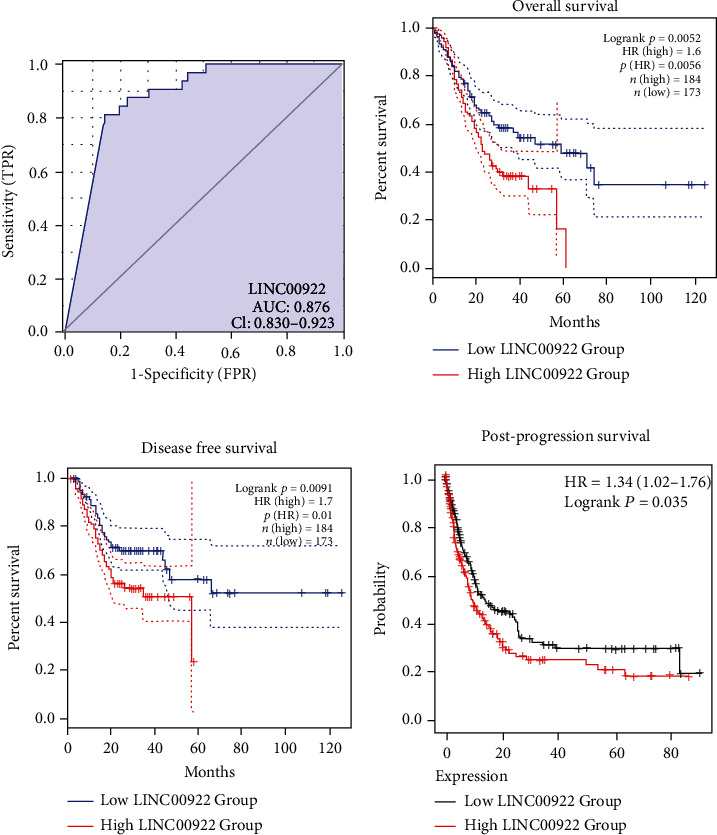
The prognostic analysis based on LINC00922 expression level in GC patients. (a) ROC curve of LINC00922 expression level in patients with GC. (b) Correlation between LINC00922 expression level and OS of patients with GC. (c) Correlation between LINC00922 expression level and DFS of GC patients. (d) Correlation between LINC00922 expression level and PPS of patients with GC (*n* = 384).

By drawing ROC curve, we analyzed whether LINC00922 had diagnostic efficacy. The area under the curve (AUC) value of LINC00922 was 0.876 (CI 0.830-0.923) (Figure 3(a)), which showed a certain accuracy, suggesting that LINC00922 could be a potential diagnostic biomarker in GC. Next, we analyzed the prognostic value of LINC00922 in GC. Through the GEPIA2 database, the survival analysis registered the high expression of LINC00922 correlated with shorter OS and DFS in GC (*n* = 357, OS: HR = 1.6, *P* = 0.0056; *n* = 357, DFS: HR = 1.7, *P* = 0.01) (Figures 3(b) and 3(c)). Then analysis via the Kaplan-Meier plotter database showed that the high expression of LINC00922 often indicated worse PPS in patients with GC (*n* = 384, HR = 1.34, *P* = 0.035) (Figure 3(d)).

Meanwhile, we also performed univariate and multivariate Cox regression analyses on the prognostic factors of GC. The univariate Cox analysis pointed to the significant correlation between the high expression of LINC00922 and poor OS (HR = 1.694, 95%CI = 1.219 − 2.355, *P* = 0.002). Multivariate regression analysis further confirmed that the expression level of LINC00922 was an independent prognostic risk factor of OS in GC patients (HR = 1.619, 95%CI = 1.120 − 2.341, *P* = 0.01) (Table 2).

### 3.4. The Analysis of LINC00922 Related Signaling Pathways

**Figure 4:**
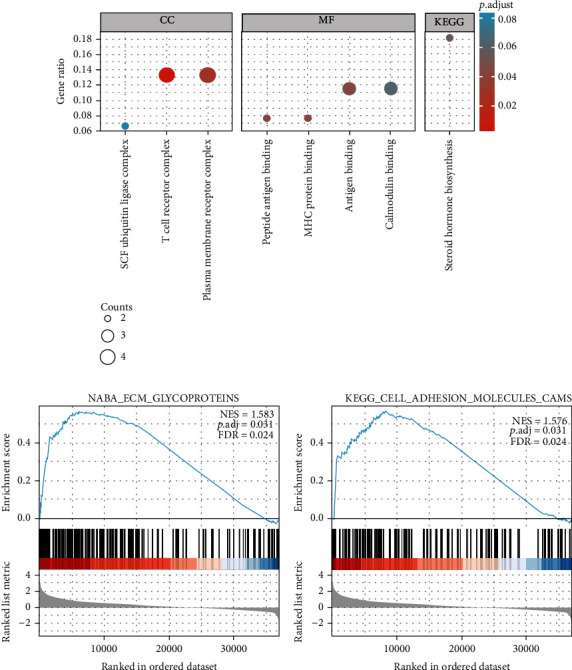
GO/KEGG enrichment analysis and GSEA signaling pathway enrichment analysis. (a) The GO/KEGG enrichment analysis results of LINC00922 coexpressed genes. The results of GSEA signaling pathway enrichment analysis: cell adhesion molecules pathway (b) and ECM glycoproteins pathway (c). (NES: normalized NS; *P*.adj: adjust *P* value; FDR: false discovery rate).

The GO enrichment analysis showed that the genes coexpressed with LINC00922 were significantly enriched in two cellular components, T cell receptor complex and plasma membrane receptor complex. The significantly enriched molecular functions were peptide antigen binding, MHC protein binding, and antigen binding. Which suggested that LINC00922 might be involved in the regulation of immune infiltration (Figure 4(a)). Then, we used the GSEA enrichment analysis to analyze the signaling pathways related to LINC00922 in GC. The results showed that two pathways related to cell adhesion (CELL_ADHESION_MOLECULES_CAMS AND ECM_GLYCOPROTEINS) had higher normalized enrichment scores (Figures 4(b) and 4(c)), suggesting that the expression of LINC00922 may be correlated with the migration and invasion of GC.

### 3.5. The Correlation between LINC00922 and Immune Infiltration in GC

**Figure 5:**
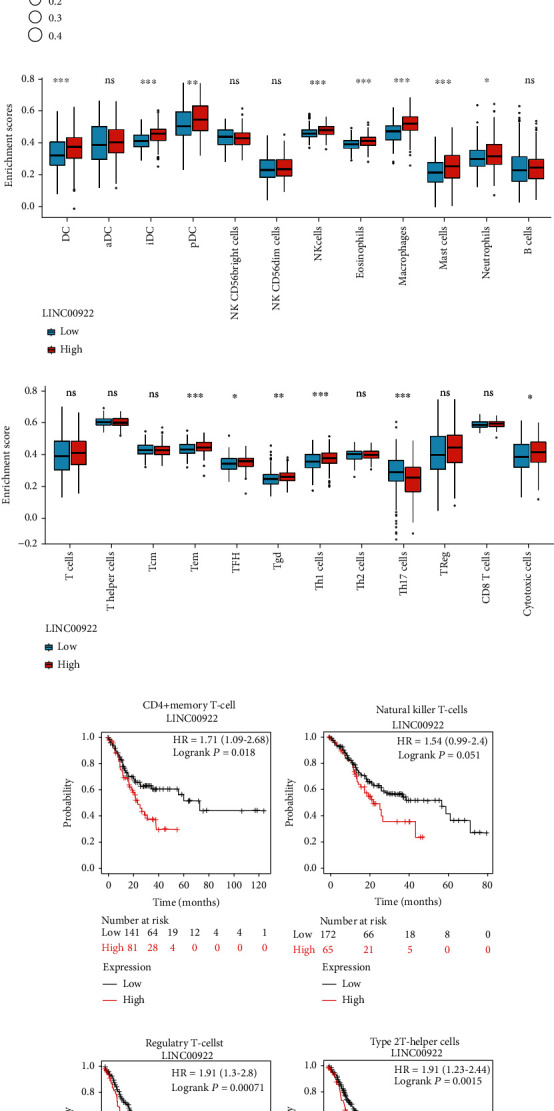
Correlation between the expression level of LINC00922 and infiltrating immune cells in GC. (a) The lollipop chart shows the correlation between LINC00922 expression level and 24 immune cells; the size of dots indicates the absolute value of the Spearman correlation coefficient. (b, c) Comparison of immune scores of GC infiltrating immune cells in a cohort of GC patients with high and low expression of LINC00922. (d)–(g) Comparison of Kaplan-Meier survival curves of LINC00922 high and low expression in CD4^+^ memory T-cells, natural killer T-cells, regulatory T-cells, and type 2 T-helper cells.

Afterward, we analyzed the correlation between the expression of LINC00922 and immune infiltration by using the ssGSEA algorithm in the GSVA package. The results showed that the expression of LINC00922 was positively correlated with the infiltration of cytotoxic cells, DC, immature DC, eosinophils, macrophages, mast cells, neutrophils, NK cells, plasmacytoid DC, T follicular helper cells, *γδ* T cells, Th1 cells, and Treg cells, while negatively correlated with infiltration of Th17 cells (Figure 5(a)). No correlation was found between LINC00922 expression and other subgroups of immune cells (Supplementary Table S3). Then, the differences of immune scores of 24 immune cells in LINC00922 high and low expression groups were analyzed by Wilcoxon rank sum test. The results showed that DC cells, immature DC cells, plasmacytoid DC, NK cells, eosinophils, macrophages, mast cells, neutrophils, T effector memory cells, T follicular helper cells, *γδ* T cells, Th1 cells, Th17 cells, and cytotoxic cells were significantly different in the high and low expression groups of LINC00922 (*P* < 0.05) (Figures 5(b) and 5(c)).

We utilized the Kaplan-Meier plotter to analyze the prognostic value of LINC00922 in the specific immune cell-enriched GC cohort. We found that in the cohort enriched with memory CD4^+^ T cells, regulatory T cells, and Th2 cells, GC patients with high expression of LINC00922 had poor prognosis (Figures 5(d), 5(f), and 5(g)). However, no significant correlation was found between LINC00922 and OS in NKT cells enrichment GC cohort (Figure 5(e)). The analyses above suggested that immune infiltration and the high expression of LINC00922 might jointly affect the prognosis of GC patients.

### 3.6. LINC00922 Promotes the Migration and Invasion of GC In Vitro

**Figure 6:**
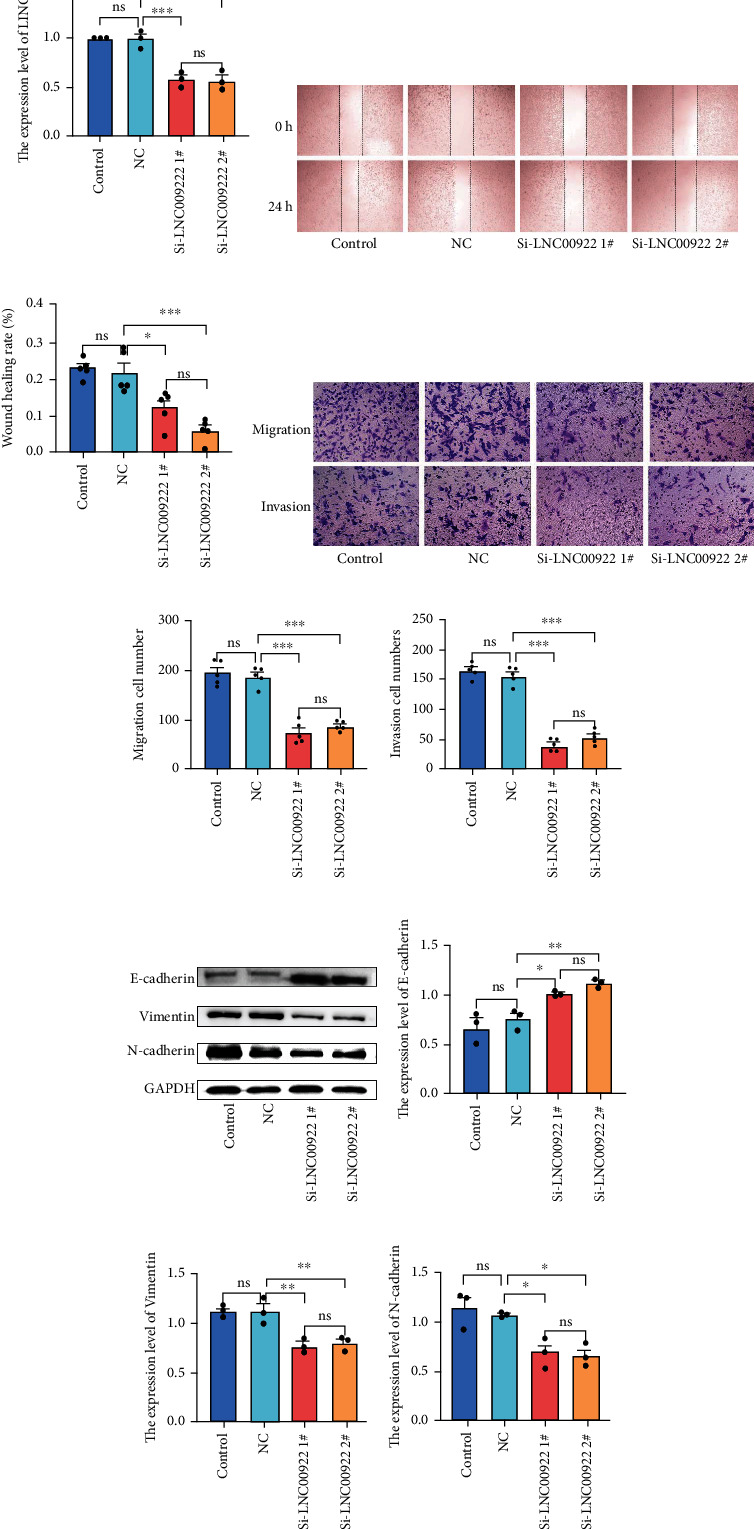
Downregulation of LINC00922 inhibited migration and invasion of GC cells. (a) qPCR analysis of LINC00922 expression. (b) Images of MKN-45 cells' healing of scored wounds. (c) The healing rate of MKN-45 cells scored wounds. (d) Migration and invasion images of MKN-45 cells in Transwell assay. (e) The number of MKN-45 cells passing through the matrix membrane in the migration assay. (f) The number of MKN-45 cells passing through the matrix membrane in the invasion assay. (g)–(j) Western blot validates the effects of LINC00922 down-regulation on the expression of Vimentin, E-cadherin, and N-cadherin.

We used qPCR to detect the expression level of LINC00922 in different GC cell lines and normal gastric mucosal cells. The expression levels of LINC00922 in MGC-803 and MKN-45 GC cell lines were significantly higher than that in normal mucosa GES-1 cell line (Supplementary Figure S1), and the expression level in MKN-45 cell line was particularly high. We, therefore, selected the MKN-45 cell line for subsequent experiments. In order to verify the effect of LINC00922 on the migration and invasion of GC cells, we designed the siRNA to downregulate the expression of LINC00922 in MKN-45 cells and then qPCR was used for confirmation. The expression levels of LINC00922 in siRNA interference groups were lower than that in the negative control group (Figure 6(a)). We then did scratch and transwell assays to evaluate the migration and invasion abilities of these cells. The results showed that compared with the control group, the downregulated groups inhibited migration and invasion of GC cells (Figures 6(b)–6(f)). Based on these results, we confirmed that the expression of LINC00922 promotes the migration and invasion of GC cells.

### 3.7. LINC00922 Promotes EMT of GC In Vitro

As was indicated, LINC00922 promoted the migration and invasion of GC cells, and based on previous studies, we hypothesized that LINC00922 promotes EMT of GC. We then detected the expression level of EMT-related proteins, including E-cadherin, vimentin, and N-cadherin. The detection showed that after downregulating LINC00922, E-cadherin, an epithelial cell marker protein, was upregulated, whereas N-cadherin and vimentin, two mesenchymal cell marker proteins, were downregulated (Figure 6(g)–6(j)). The above results indicated that LINC00922 could promote EMT of GC cells.

### 3.8. LINC00922 Promotes the Growth, Migration, and Invasion of GC In Vivo

**Figure 7:**
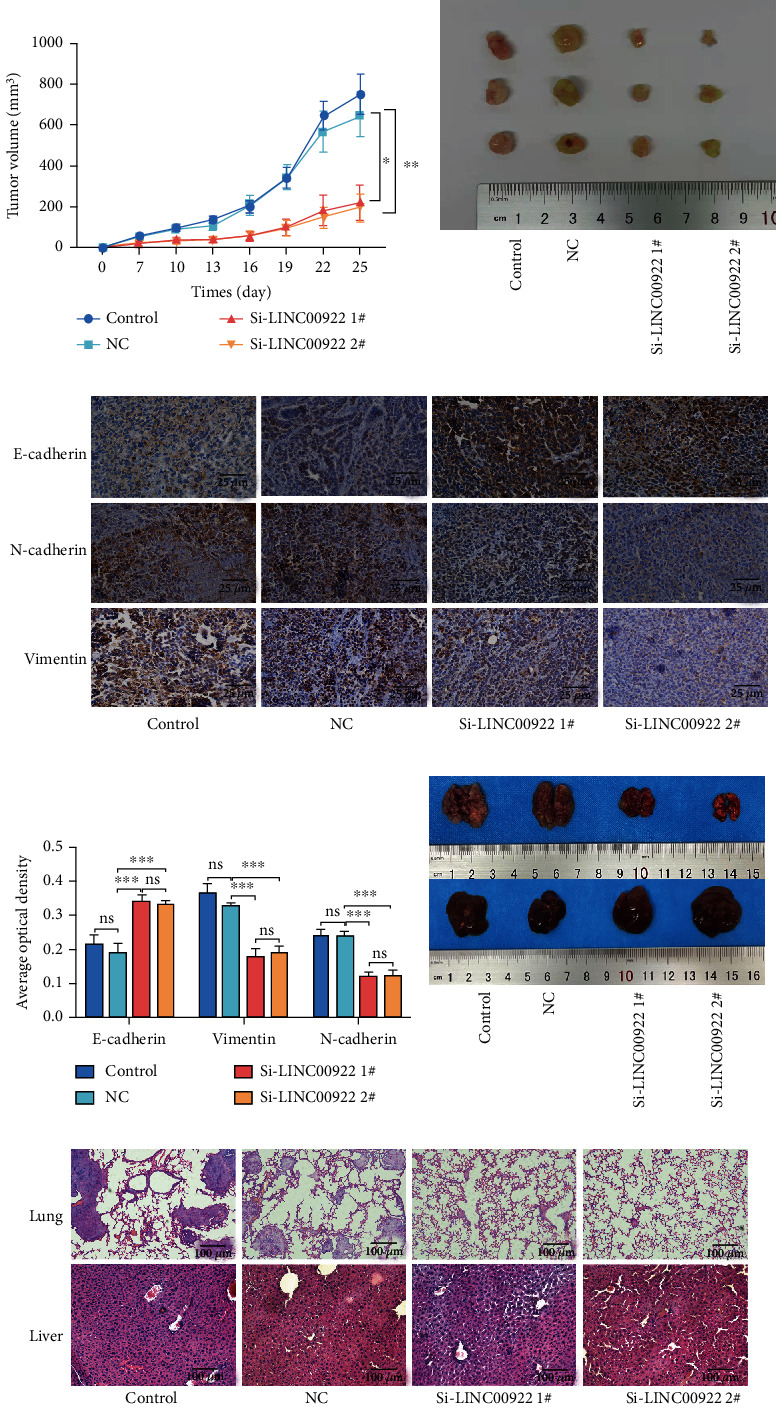
Downregulation of LINC00922 inhibited growth, migration, and invasion of GC in vivo. (a) Growth curves of xenograft tumors of four groups. (b) Representative images of xenograft tumors of four groups. (c) Representative images of the expression levels of E-cadherin, vimentin, and N-cadherin in each group were assessed by immunohistochemistry (×400). (d) The expression levels of E-cadherin, vimentin, and N-cadherin were analyzed by one-way ANOVA in each group. (e) Representative images of lung metastasis and spleen in nude mice of each group. (f) The lung and liver metastasis levels in nude mice of four groups were assessed by HE staining (×400).

In order to further explore the biological correlation of LINC00922 on the growth and metastasis of GC in vivo, we constructed the xenograft tumor model and metastasis tumor model in nude mice. We found that the growth of tumors in the LINC00922 downregulated groups was significantly delayed compared with the control group and the negative control group (Figures 7(a) and 7(b)). Immunohistochemical staining showed that after the downregulation of LINC00922, the expression level of E-cadherin increased, while vimentin and N-cadherin decreased (Figures 7(c) and 7(d)). It is worth noting that downregulation of LINC00922 reduced the metastasis efficiency of GC cells to lung in nude mice, but no liver metastasis was detected in any group (Figures 7(e) and 7(f)). These results suggested that LINC00922 can promote the growth, EMT, and metastasis of GC in vivo.

## 4. Discussion

Gastric cancer is one of the most common malignancies of the gastrointestinal tract, with high morbidity and mortality [1]. The occurrence of GC is a multifactorial and multistep complex process. Previous studies have shown that lncRNAs are involved in gastric carcinogenesis and development by exerting a regulatory role in gene expression at the epigenetic, transcriptional, or posttranscriptional levels [30]. The expression of lncRNA is tightly regulated, and its expression patterns exhibit temporal and spatial specificity [31]. Dysregulated lncRNAs are considered to be a useful metric for characterizing the molecular profile of GC, affecting patients' prognosis through multiple molecular pathways [32, 33]. Therefore, exploring dysregulated molecular signatures that are upregulated or downregulated in GC would be available for diagnosis, treatment, and prognosis [34].

Increasing evidence has illustrated that LINC00922 was highly expressed in various types of cancer, including ovarian cancer [22] and colorectal cancer [35]. For example, Wang et al. [36] found that LINC00922 was highly expressed in ovarian cancer cell lines, while Zhang et al. [35] reported that LINC00922 was highly expressed in colorectal tissues and cell lines. These results are similar to the present study, and the expression of LINC00922 was significantly higher in GC cell lines. In addition, the prognostic significance of LINC00922 expression has been investigated in lung cancer [36], ovarian cancer [22], and bladder cancer [18]. For instance, Liang et al. revealed that patients with high LINC00922 expression had unfavorable prognosis than those with low LINC00922 expression in lung cancer [36]. The prognostic significance of LINC00922 expression in GC has remained unknown until now. We first found LINC00922 expression was negatively linked with clinical outcomes in GC patients in the TCGA database or our investigation. These results suggested that LINC00922 may function as an oncogene in the initiation and progression of GC.

Metastasis to adjacent or distant organs is the primary cause of mortality in patients with GC [10]. Cell motility and invasive behavior of gastric cancer cells is the critical mechanism underlying cancer metastasis [37]. Previous studies indicate that LINC00922 participates in metastasis regulation of breast cancer [23] and colorectal cancer [35]. In this study, we designed short hairpin RNA to interfere with the expression of LINC00922 in GC cells and performed scratch and transwell assays. The results showed that the downregulation of LINC00922 in GC cells reduced the number of cells that migrated and invaded. It is worth noting that the downregulation of LINC00922 decreased the number and diameter of metastatic nodules colonized of the lungs in the tumor metastasis model of nude mice. Therefore, we obtained a direct indication that manipulation of LINC00922 expression level affects the invasion and migration abilities of GC cells.

EMT, a phenomenon in which epithelial cells lose cell polarity and obtain a migratory mesenchymal phenotype, is a vital biological process inducing tumor invasion and metastasis [38]. Simultaneously, this cellular event is characterized by loss of E-cadherin, an epithelial cell marker, and increase of mesenchymal markers such as N-cadherin and Vimentin [39]. Accumulating evidence demonstrated that lncRNAs regulate GC cell invasion and metastasis by inducing EMT process [40]. It has been proved that LINC00922 played an important role in promoting EMT in ovarian cancer [22], breast cancer [23], and liver cancer [21]. In the present study, we found that the expression of E-cadherin was upregulated by downregulating the expression of LINC00922, while the expressions of the corresponding mesenchymal cell markers vimentin and N-cadherin were significantly decreased. Which indicated that the expression of LINC00922 in GC cells could induce the EMT process.

However, the results of our study are limited to functional studies. At present, further experiments are needed to explore the specific molecular mechanism of LINC00922 regulating the invasion, migration, and EMT process of GC cells.

In conclusion, the present study demonstrated that LINC00922 was significantly upregulated in GC tissues and cell lines, and high LINC00922 expression predicted the much poorer prognosis of GC patients. In addition, LINC00922 knockdown inhibited migration, invasion, and EMT process of GC cells, suggesting that LINC00922 may be used as a potential target for treating patients with GC.

## Figures and Tables

**Table 1:** The expression of LINC00922 associated with clinicopathologic characteristics of GC by logistic regression analysis.

Characteristics	Total (*N*)	Odds ratio (OR)	*P* value
T stage (T2&T3&T4 vs. T1)	367	8.900 (2.502-56.673)	0.004
N stage (N2&N3 vs. N0&N1)	357	0.900 (0.590-1.371)	0.623
M stage (M1 vs. M0)	355	1.821 (0.798-4.414)	0.164
Primary therapy outcome (SD&PR vs. CR&PD)	317	1.762 (0.721-4.569)	0.222
Race (Asian and Black or African American vs. White)	323	0.510 (0.305-0.841)	0.009
Histological type (papillary type and signet ring type and tubular type vs. diffuse type and mucinous type and not otherwise specified)	374	0.327 (0.191-0.544)	<0.001
Histologic grade (G3 vs. G1&G2)	366	1.677 (1.102-2.563)	0.016

CR: complete response; PD: progressive disease; SD: stable disease; PR: partial response.

**Table 2:** Univariate and multivariate Cox regression analysis of OS in patients with GC.

Characteristics	Total (*N*)	Univariate analysis		Multivariate analysis	
		Hazard ratio (95% CI)	*P* value	Hazard ratio (95% CI)	*P* value
T stage	362				
T1	18	Reference			
T2	78	6.725 (0.913-49.524)	0.061	4.366 (0.555-34.337)	0.161
T3	167	9.548 (1.326-68.748)	0.025	5.318 (0.610-46.393)	0.131
T4	99	9.634 (1.323-70.151)	0.025	4.643 (0.516-41.784)	0.171
N stage	352				
N0	107	Reference			
N1	97	1.629 (1.001-2.649)	0.049	1.291 (0.638-2.613)	0.477
N2	74	1.655 (0.979-2.797)	0.060	1.457 (0.613-3.467)	0.394
N3	74	2.709 (1.669-4.396)	<0.001	2.058 (0.865-4.894)	0.103
M stage	352				
M0	327	Reference			
M1	25	2.254 (1.295-3.924)	0.004	1.197 (0.505-2.838)	0.683
Race	320				
Asian	73	Reference			
Black or African American	11	1.949 (0.808-4.698)	0.137		
White	236	1.449 (0.873-2.405)	0.152		
Age	367				
≤65	163	Reference			
>65	204	1.620 (1.154-2.276)	0.005	1.859 (1.278-2.704)	0.001
Gender	370				
Female	133	Reference			
Male	237	1.267 (0.891-1.804)	0.188		
Pathologic stage	347				
Stage I	50	Reference			
Stage II	110	1.551 (0.782-3.078)	0.209	1.086 (0.383-3.075)	0.877
Stage III	149	2.381 (1.256-4.515)	0.008	1.080 (0.272-4.290)	0.913
Stage IV	38	3.991 (1.944-8.192)	<0.001	2.044 (0.502-8.327)	0.318
Histologic grade	361				
G1	10	Reference			
G2	134	1.648 (0.400-6.787)	0.489		
G3	217	2.174 (0.535-8.832)	0.278		
LINC00922	370	1.694 (1.219-2.355)	0.002	1.619 (1.120-2.341)	0.010

## Data Availability

The data used to support the bioinformatics analysis of this study are available from the TCGA database (https://portal.gdc.cancer.gov/) and UCSC Xena database (https://xenabrowser.net/datapages/). The raw data of the experiments are available from the corresponding author upon request.

## Supplementary Materials

Supplementary Materials Supplementary Figure 1: expression of LINC00922 in GES-1, MGC-803, and MKN-45 cell lines. Supplementary Table S1: the correlation between LINC00922 expression and clinicopathological characteristics in GC. Supplementary Table S2: the primers and siRNA sequences. Supplementary Table S3: correlation between LINC00922 and infiltrating immune cells in GC.
