# Supplementary material for: Upregulated LINC00922 Promotes Epithelial-Mesenchymal Transition and Indicates a Dismal Prognosis in Gastric Cancer
Source: J Oncol. 2022 Apr 11;2022:1608936. doi: 10.1155/2022/1608936 (PMC9015875; doi:10.1155/2022/1608936)
Supplement: Supplementary Materials — Supplementary Figure 1: expression of LINC00922 in GES-1, MGC-803, and MKN-45 cell lines. Supplementary Table S1: the correlation between LINC00922 expression and clinicopathological characteristics in GC. Supplementary Table S2: the primers and siRNA sequences. Supplementary Table S3: correlation between LINC00922 and infiltrating immune cells in GC. [file 1608936.f1.zip › Supplementary Table S1. The correlation between LINC00922 expression and clinicopathological characteristics in GC.docx]

Table 1. The correlation between LINC00922 expression and clinicopathological characteristics in GC.

| Characteristic | Low expression of LINC00922 | High expression of LINC00922 | p |
| --- | --- | --- | --- |
| N | 187 | 188 |  |
| T stage, n (%) |  |  | **0.004** |
| T1 | 17 (4.6%) | 2 (0.5%) |  |
| T2 | 36 (9.8%) | 44 (12%) |  |
| T3 | 88 (24%) | 80 (21.8%) |  |
| T4 | 46 (12.5%) | 54 (14.7%) |  |
| N stage, n (%) |  |  | 0.955 |
| N0 | 55 (15.4%) | 56 (15.7%) |  |
| N1 | 47 (13.2%) | 50 (14%) |  |
| N2 | 38 (10.6%) | 37 (10.4%) |  |
| N3 | 39 (10.9%) | 35 (9.8%) |  |
| M stage, n (%) |  |  | 0.230 |
| M0 | 167 (47%) | 163 (45.9%) |  |
| M1 | 9 (2.5%) | 16 (4.5%) |  |
| Gender, n (%) |  |  | 0.251 |
| Female | 61 (16.3%) | 73 (19.5%) |  |
| Male | 126 (33.6%) | 115 (30.7%) |  |
| Primary therapy outcome, n (%) |  |  | 0.060 |
| PD | 40 (12.6%) | 25 (7.9%) |  |
| SD | 8 (2.5%) | 9 (2.8%) |  |
| PR | 0 (0%) | 4 (1.3%) |  |
| CR | 114 (36%) | 117 (36.9%) |  |
| Race, n (%) |  |  | **0.022** |
| Asian | 44 (13.6%) | 30 (9.3%) |  |
| Black or African American | 8 (2.5%) | 3 (0.9%) |  |
| White | 106 (32.8%) | 132 (40.9%) |  |
| Age, n (%) |  |  | 0.771 |
| <=65 | 79 (21.3%) | 85 (22.9%) |  |
| >65 | 104 (28%) | 103 (27.8%) |  |
| Histological type, n (%) |  |  | **0.001** |
| Diffuse Type | 30 (8%) | 33 (8.8%) |  |
| Mucinous Type | 9 (2.4%) | 10 (2.7%) |  |
| Not Otherwise Specified | 88 (23.5%) | 119 (31.8%) |  |
| Papillary Type | 3 (0.8%) | 2 (0.5%) |  |
| Signet Ring Type | 7 (1.9%) | 4 (1.1%) |  |
| Tubular Type | 50 (13.4%) | 19 (5.1%) |  |
| Histologic grade, n (%) |  |  | **0.049** |
| G1 | 5 (1.4%) | 5 (1.4%) |  |
| G2 | 79 (21.6%) | 58 (15.8%) |  |
| G3 | 97 (26.5%) | 122 (33.3%) |  |
| Age, mean ± SD | 65.61 ± 10.32 | 66.05 ± 10.99 | 0.690 |

CR, complete response; PD, progressive disease; SD, stable disease; PR, partial response.
