# Supplementary material for: Upregulated LINC00922 Promotes Epithelial-Mesenchymal Transition and Indicates a Dismal Prognosis in Gastric Cancer
Source: J Oncol. 2022 Apr 11;2022:1608936. doi: 10.1155/2022/1608936 (PMC9015875; doi:10.1155/2022/1608936)
Supplement: Supplementary Materials — Supplementary Figure 1: expression of LINC00922 in GES-1, MGC-803, and MKN-45 cell lines. Supplementary Table S1: the correlation between LINC00922 expression and clinicopathological characteristics in GC. Supplementary Table S2: the primers and siRNA sequences. Supplementary Table S3: correlation between LINC00922 and infiltrating immune cells in GC. [file 1608936.f1.zip › Supplementary Table S2. The primers and siRNA sequences.docx]

Supplementary Table S2: The primers and siRNA sequences.

| Primer/siRNA | sequences |
| --- | --- |
| LINC00922-F | ATAGGAAACCCAAAGGGAACA |
| LINC00922-R | GAGGAGCAGCAGGGAAGAAA |
| GAPDH-F | ACAACTTTGGTATCGTGGAAGG |
| GAPDH-R | GCCATCACGCCACAGTTTC |
| Si-LINC00922-1 | GCCCAUUCCAGAAUUUACUTT |
| Si-LINC00922-2 | CAGGCUACAAUCCUUUAAATT |
