# Supplementary material for: Upregulated LINC00922 Promotes Epithelial-Mesenchymal Transition and Indicates a Dismal Prognosis in Gastric Cancer
Source: J Oncol. 2022 Apr 11;2022:1608936. doi: 10.1155/2022/1608936 (PMC9015875; doi:10.1155/2022/1608936)
Supplement: Supplementary Materials — Supplementary Figure 1: expression of LINC00922 in GES-1, MGC-803, and MKN-45 cell lines. Supplementary Table S1: the correlation between LINC00922 expression and clinicopathological characteristics in GC. Supplementary Table S2: the primers and siRNA sequences. Supplementary Table S3: correlation between LINC00922 and infiltrating immune cells in GC. [file 1608936.f1.zip › Supplementary Table S3. Correlation between LINC00922 and infiltrating immune cells in GC.docx]

Supplementary Table S3. Correlation between LINC00922 and infiltrating immune cells in GC.

| Immune cells | Spearman correlation | *P* value |
| --- | --- | --- |
| aDC (activated DC) | 0.092 | 0.075 |
| B cells | 0.04 | 0.442 |
| CD8^+^ T cells | 0.097 | 0.06 |
| Cytotoxic cells | 0.165 | **0.001** |
| DC (Dendritic Cells) | 0.224 | **<0.001** |
| Eosinophils | 0.246 | **<0.001** |
| iDC (immature DC) | 0.352 | **<0.001** |
| Macrophages | 0.436 | **<0.001** |
| Mast cells | 0.193 | **<0.001** |
| Neutrophils | 0.12 | **0.02** |
| NK CD56bright cells | -0.073 | 0.159 |
| NK CD56dim cells | 0.083 | 0.109 |
| NK cells | 0.328 | **<0.001** |
| pDC (plasmacytoid DC) | 0.183 | **<0.001** |
| T cells | 0.114 | **0.028** |
| T helper cells | -0.036 | 0.489 |
| Tcm (T central memory cells) | -0.027 | 0.6 |
| Tem (T effector memory cells) | 0.191 | **<0.001** |
| TFH (T follicular helper cells) | 0.113 | **0.028** |
| Tgd (γδ T cells) | 0.162 | **0.002** |
| Th1 cells | 0.216 | **<0.001** |
| Th17 cells | -0.24 | **<0.001** |
| Th2 cells | 0.011 | 0.826 |
| Treg (regulatory T cells) | 0.128 | **0.013** |
