# Supplementary figures and images for: Upregulated LINC00922 Promotes Epithelial-Mesenchymal Transition and Indicates a Dismal Prognosis in Gastric Cancer
Source: J Oncol. 2022 Apr 11;2022:1608936. doi: 10.1155/2022/1608936 (PMC9015875; doi:10.1155/2022/1608936)

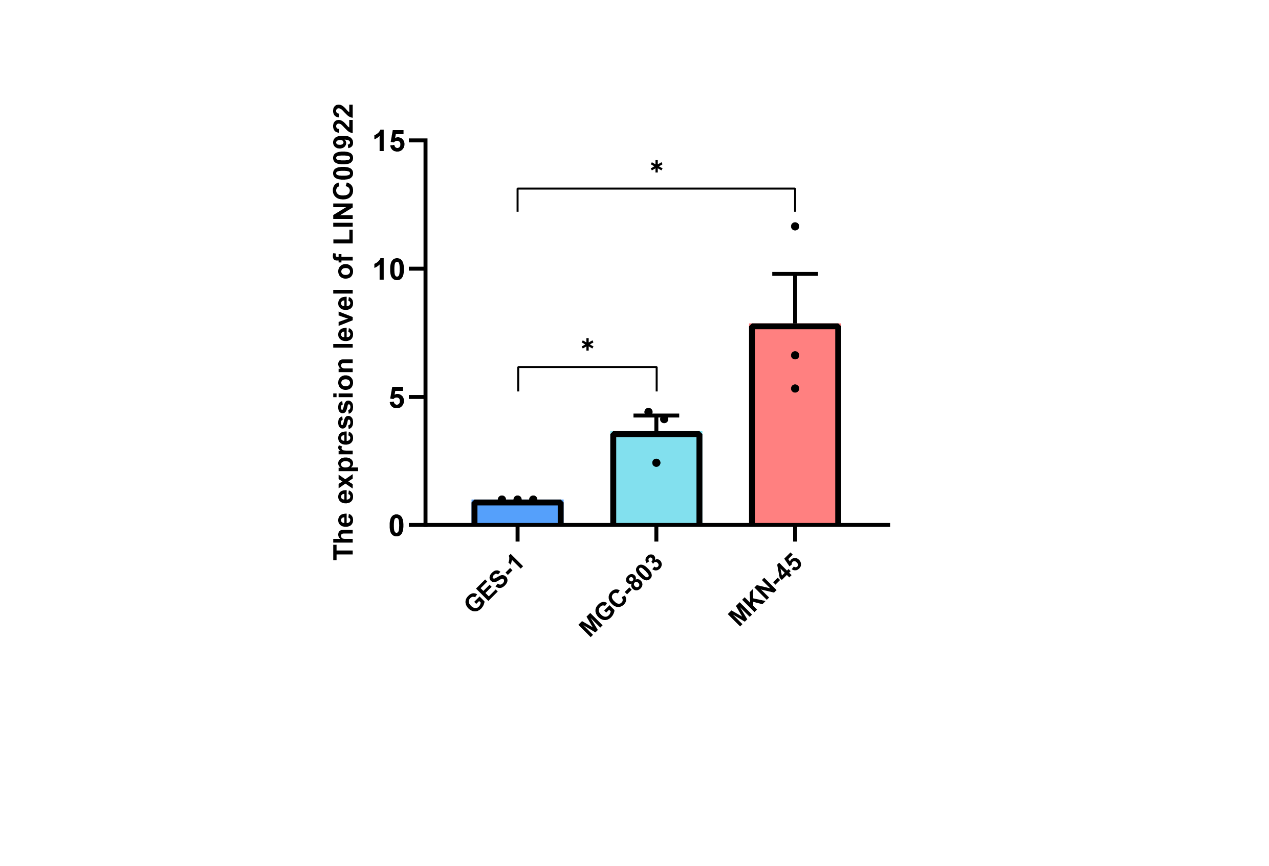
Supplementary Figure 1. Expression of LINC00922 in GES-1, MGC-803, MKN-45 cell lines.

Supplement: Supplementary Materials — Supplementary Figure 1: expression of LINC00922 in GES-1, MGC-803, and MKN-45 cell lines. Supplementary Table S1: the correlation between LINC00922 expression and clinicopathological characteristics in GC. Supplementary Table S2: the primers and siRNA sequences. Supplementary Table S3: correlation between LINC00922 and infiltrating immune cells in GC. [file 1608936.f1.zip › Supplementary Figure 1.Expression of LINC00922 in GES-1, MGC-803, MKN-45 cell lines.docx]
